# Expression Pattern of α-Tubulin, Inversin and Its Target Dishevelled-1 and Morphology of Primary Cilia in Normal Human Kidney Development and Diseases

**DOI:** 10.3390/ijms22073500

**Published:** 2021-03-28

**Authors:** Ivana Solic, Anita Racetin, Natalija Filipovic, Snjezana Mardesic, Ivana Bocina, Danica Galesic-Ljubanovic, Meri Glavina Durdov, Mirna Saraga-Babić, Katarina Vukojevic

**Affiliations:** 1 Department of Anatomy, Histology and Embryology, School of Medicine, University of Split, Šoltanska 2, 21000 Split, Croatia; ivana.solic@mefst.hr (I.S.); anita.muic@mefst.hr (A.R.); natalija.filipovic@mefst.hr (N.F.); snjezana.mardesic.brakus@mefst.hr (S.M.); msb@mefst.hr (M.S.-B.); 2 Department of Medical Genetics, School of Medicine, University of Mostar, 88000 Mostar, Bosnia and Herzegovina; 3 Department of Biology, Faculty of Science, University of Split, 21000 Split, Croatia; ivana.bocina@pmfst.hr; 4 Department of Pathology, Clinical Hospital Dubrava, 10000 Zagreb, Croatia; danica.ljubanovic@mef.hr; 5 Department of Pathology, University of Zagreb School of Medicine, 10000 Zagreb, Croatia; 6 Department of Pathology, University Hospital of Split, 21000 Split, Croatia; merigdst@yahoo.co.uk; 7 Department of Pathology, School of Medicine, University of Split, Šoltanska 2, 21000 Split, Croatia

**Keywords:** human kidney development, α-tubulin, inversin, DVL-1, MCDK, FSGS, CNF

## Abstract

The spatiotemporal expression of α-tubulin, inversin and dishevelled-1 (DVL-1) proteins associated with the Wnt-signaling pathway, and primary cilia morphology were analyzed in developing kidneys (14th–38th developmental weeks), healthy postnatal (1.5- and 7-years old) and pathologically changed human kidneys, including multicystic dysplastic kidneys (MCDK), focal segmental glomerulosclerosis (FSGS) and nephrotic syndrome of the Finnish type (CNF). The analysis was performed by double immunofluorescence, electron microscopy, semiquantitative and statistical methods. Cytoplasmic co-expression of α-tubulin, inversin and DVL-1 was observed in the proximal convoluted tubules (pct), distal convoluted tubules (dct) and glomeruli (g) of analyzed tissues. During kidney development, the overall expression of α-tubulin, inversin and DVL-1 decreased, while in the postnatal period slightly increased. The highest expressions of α-tubulin and inversin characterized dct and g, while high DVL-1 characterized pct. α-tubulin, inversin and DVL-1 expression pattern in MCDK, FSGS and CNF kidneys significantly differed from the healthy control. Compared to healthy kidneys, pathologically changed kidneys had dysmorphic primary cilia. Different expression dynamics of α-tubulin, inversin and DVL-1 during kidney development could indicate that switch between the canonical and noncanonical Wnt-signaling is essential for normal kidney morphogenesis. In contrast, their disturbed expression in pathological kidneys might be associated with abnormal primary cilia, leading to chronic kidney diseases.

## 1. Introduction

The development of the definitive or metanephric kidneys begins during the fifth gestational week (GW), which then continuously differentiates to form the permanent kidneys [1,2]. The signaling interactions between the metanephric mesenchyme and the ureteric bud ensure the proper kidney development during nephrogenesis. Briefly, the ureteric bud induces mesenchymal-to-epithelial transition (MET) in the metanephric mesenchyme, which condensates and forms renal vesicles, followed by comma-shaped and S-shaped bodies, and finally leading to glomeruli formation [3]. In return, the mesenchyme induces further branching of the ureteric bud. The metanephric kidneys become functional excretory units at the 11th week of human development. However, nephrogenesis is completed within the 34th to 36th week of fetal development, when multiple branching events are finished, but further differentiation process of kidneys continues into the postnatal period [4,5]. Disruptions of these complex interactions result in various congenital abnormalities of the kidney and urinary tract (CAKUT), including dysplasia, polycystic kidney disease, multicystic dysplastic kidney disease (MCDK), consequently leading to chronic kidney disease (CKD) [6].

In this study, we were focused on primary cilia appearance and the Wnt-signaling pathway, which plays an important role during normal nephrogenesis and in the kidney repair process, following acute or chronic kidney disease [7]. The existence of primary cilia during nephrogenesis and the large number of developmental kidney defects occurring in patients with ciliary disease point out that genuine primary cilia function is necessary for normal kidney organogenesis [8,9]. The primary cilium is a microtubule-based organelle important for tissue homeostasis, where α-tubulin is the basic component [10]. Loss of α-tubulin acetylation in immortalized cells triggers the epithelial-to-mesenchymal transition (EMT), thus implying that acetylated α-tubulin is important in the stabilization of microtubules [11]. During human development, the primary cilia-mediated Wnt pathway enables cell proliferation, differentiation and tissue morphogenesis [12]. A significant number of children with impaired primary cilia function and disrupted Wnt-signaling pathway develop CKD, where congenital renal disorders are responsible for almost half of the cases [13]. The most common congenital cystic disease in children is multicystic dysplastic kidney disease (MCDK) [14]. Multiple cysts that do not communicate [15] and lack normal renal parenchyma are characteristic microscopic findings for MCDK. Focal segmental glomerulosclerosis (FSGS) is one of the most common glomerular diseases that lead to end-stage kidney disease [16]. In the beginning, FSGS is characteristic of being focal, involving only a minority of the glomeruli and segmental, involving changes in only a segment of the glomerular circle [17]. Congenital nephrotic syndrome of the Finnish type (CNF) is an uncommon autosomal recessively inherited kidney disease represented by a prenatal outbreak of extensive protein loss [18], associated with cystogenesis of proximal tubules [19]. Many forms of primary glomerular diseases develop proteinuria as a result of the damaged glomerular filtration barrier [20]. A considerable amount of studies implies that injury and dysfunction of the podocytes have intrinsic roles in the pathogenesis of CKD proteinuria [21]. The study by Vukojevic et al. shows an increased number of ciliated and poorly differentiated podocytes in CNF [22]. Previous studies indicated that the Wnt/β-catenin signaling pathway has a fundamental role in moderating podocyte dysfunction with proteinuria [23]. There are two main Wnt-signaling pathways, one known as canonical β-catenin-dependent and other noncanonical, β-catenin independent. During the mouse kidney development, canonical Wnt-signaling is active on the ureteric bud tips and in S-shaped bodies [24]. Furthermore, Wnt via canonical signaling has relevance in maintaining MET during nephrogenesis [25]. Namely, binding of the protein complex containing Dvl to the membrane receptor is mandatory for Wnt pathway activation, while disabling the key components of this pathway results in early perinatal mortality due to the absence of the nephrogenic zone in the kidney [25].

A protein crucial as a molecular switch between canonical and noncanonical Wnt-signaling pathways was found to be inversin [26]. Although interactions of various proteins with inversin have been detected, its exact function has not yet been completely clarified. In the renal epithelial cells, inversin is located in the primary cilia, acting as a ciliary protein [27]. In addition, inversin forms a steady complex with tubulin in cultures of renal cells, and they colocalize in vivo [27,28]. Inversin seems to play an essential role in the early morphogenesis of the pronephric system and left-right symmetry determination during development [29,30].

A significant event for both canonical and noncanonical Wnt-signaling pathways in the recruitment of the cytoplasmic Dvl to the cell membrane. Since previous findings showed that inversin and Dvl colocalize in the renal epithelial cells, it can be speculated that inversin might also have a role in noncanonical signaling. Dysregulation of Wnt-signaling mediated by inversin triggers aberrant proliferation in tubules [31]. Genetic tests revealed a DVL-1 mutation in patients with autosomal-dominant Robinow syndrome, presenting heterogeneous disorders in some patients associated with urogenital and renal anomalies [32].

The developmental expression pattern of α-tubulin, inversin and DVL-1 has been investigated in different animal models. Thus, their expression in pronephros of *Xenopus laevis* has been confirmed by using intravital microscopy [31], while they were found in cell lines derived from murine proximal tubular cells by using confocal microscopy and mass spectrometry [33]. In addition, light microscopy and double immunofluorescence revealed their presence in sections of mice kidneys [34]. Studies on inversin knockout mice reported enlarged, diffused cysts in the renal medulla and cortex associated with situs viscerum inversus [35]. In humans, mutation of inversin was presented with infantile nephronophthisis that led to the termination of pregnancy [36].

Despite numerous studies on renal α-tubulin, inversin and DVL-1 expression pattern and function, to the best of our knowledge, an expression pattern of these proteins in the human fetal and postnatal human tissue has not been investigated yet and brought about to a correlation. In addition, there has not been reported evidence considering the expression of inversin and DVL-1 in early human development. Therefore, this study aimed to analyze the spatial and temporal expression pattern of α-tubulin, inversin and DVL-1 during fetal and postnatal stages of heathy human kidneys, as well as in kidney tissues of MCDK, FSGS and CNF kidneys in order to establish a possible missing link between these entities. Namely, we propose that disturbed expression patterns of α-tubulin, inversin and DVL-1 proteins might be the underlying cystogenesis process and an abnormal function of primary cilia leading to chronic kidney diseases.

## 2. Results

The analysis of developmental and postnatal kidney tissues was performed on distinctly differentiated structures of the kidney: proximal convoluted tubules (pct), distal convoluted tubules (dct) and glomeruli (g). During the analyzed developmental stages and in postnatal kidney tissues, α-tubulin, inversin and DVL-1 showed positive expression patterns but with differences in intensity, distribution and quantity. The analysis of the pathological kidney tissue of MCDK, FSGS and CNF was performed on full image count of the positive cells compared to healthy kidney tissue control.

### 2.1. Light Microscopy, Electron Microscopy and Immunohistochemistry (α-Tubulin) of Healthy and Pathologically Changed Postnatal Human Kidney Tissue

#### 2.1.1. Healthy Postnatal Kidney

**Figure 1:**
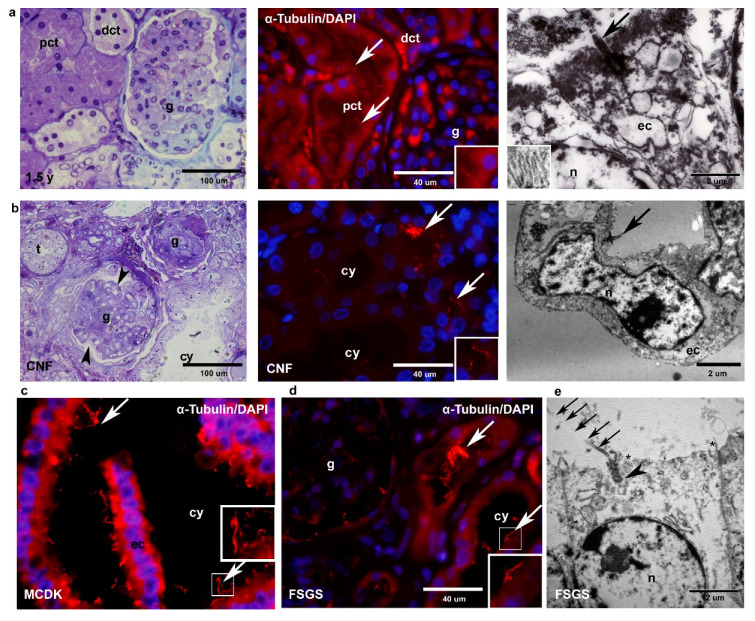
Toluidine blue staining of semi-thin sections, immunofluorescence staining with α-tubulin and 4’,6-diamidino-2-phenylindole dihydrochloride (DAPI), and transmission electron microscopy of the cortex of healthy 1.5-year-old kidney tissue (**a**) and nephrotic syndrome of the Finnish type (CNF) kidney tissue (**b**). Details of microvilli in healthy kidney tissue at the apical surface are shown as higher magnification inset (row **a**, electron microscopy). Legend: proximal convoluted tubules (pct), distal convoluted tubules (dct), glomeruli (g), tubules (t), proximal tubules cyst (cy), nucleus (n), epithelial cell (ec), primary cilium (arrows), lobulated glomerulus (arrowhead). Immunofluorescence staining with α-tubulin and DAPI shows dysmorphic primary cilia in multicystic dysplastic kidneys (MCDK) (**c**) and focal segmental glomerulosclerosis (FSGS) (**d**). Electron microscopy of FSGS distal tubular cell with extremely long primary cilium (arrows) at the apical surface (**e**). Nucleus (n), basal body (arrowhead), loss of microvilli on the apical surface (*). Details of primary cilia are shown as higher magnification insets. Magnification toluidine blue sections: ×40, scale bar 100 µm. Magnification immunofluorescence staining: ×100, scale bar 40 µm. Scale bar transmission electron microphotos 2 µm.

Light microscopy of the postnatal kidney tissue demonstrates well-defined kidney structures, with a distinct difference between medulla and cortex and the formation of the pct, dct and g in the cortical region. Immunohistochemical staining of α-tubulin reveals the presence of one primary cilium in the center of the apical cell surface of each tubular cell and in 0.018% ± 0.00014% SD on the surface of glomerular cells. Electron microscopy shows the presence of only one primary cilium arising from the basal body at the apical cell surface of the tubule (Figure 1a).

#### 2.1.2. CNF

Most of the analyzed g are smaller and lobulated (80%), while the minority are of normal size or hypertrophic (20%, *n* = 100). Segmental or global sclerosis of g is found, while pct and dct are partially dilated and coated with atrophic epithelium. Ultrastructurally, loss of microvilli and decrease in cell height with multifragmentation of nuclei is observed. In podocytes, foot processes are diffusely lost. Tubular primary cilia are disorganized, particularly in the proximal tubules’ cysts, showing changes in length and cytoplasmic position (Figure 1b).

#### 2.1.3. MCDK

In renal tissue, sporadic immature and mature g can be found. Kidney tissue is filled with numerous oval cysts. Columnar epithelial cells cover the cysts and are surrounded by loose connective tissue. Immunohistochemical staining to α-tubulin shows multiple long and disorganized primary cilia on the surface of the epithelial cells (Figure 1c).

#### 2.1.4. FSGS

In 90% of g, extensive segmental sclerosis is found (*n* = 100), associated with interstitial fibrosis and signs of tubular damage. The decrease in the height of epithelial cells with loss of microvilli is found in the electron microscope, while endothelial cells and mesangial regions have regular ultrastructure. In the electron microscope, the extremely long and dislocated (decentralized) cilium is observed on the surface of the distal tubular cell (Figure 1e). Podocytes show diffuse loss of foot processes with extensive microvillus transformation. The primary cilia appear extremely long, while multiple cilia are observed in g, pct and dct (Figure 1d).

### 2.2. Immunohistochemical Staining to α-Tubulin, Inversin and DVL-1- and Statistical Analysis of Developing and Healthy Postnatal Human Kidneys

**Figure 2:**
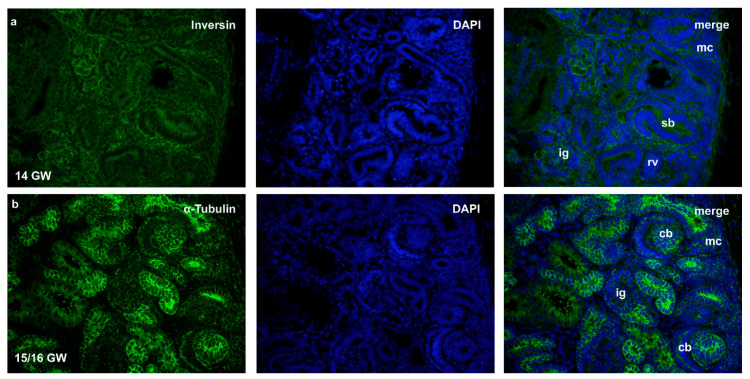
Immunofluorescence staining of inversin (**a**) and α-tubulin (**b**) in developing kidney tissues (14th and 15th/16th gestational week (GW)) showing nephrogenic zone with stages of development during the formation of the nephron: metanephric cup (mc), renal vesicle (rv), comma-shaped body (cb), S-shaped body (sb), immature glomerulus (ig). Magnification ×40, scale bar 100 µm.

In the 14th, 15th and 16th gestational weeks, the kidney tissue is presented with different developmental stages of nephron formation: from metanephric cups, renal vesicles stages to comma and S-shaped nephrons, thus forming the nephrogenic zone in the outer cortex (Figure 2). Differentiating pct, dct and g can be observed in cortical regions closer to the kidney medulla. During the 22nd GW, parts of medulla and cortex became clearly distinctive, while in 38th GW, kidney structures appear as highly differentiated, containing mature forms of pct, dct and g.

#### 2.2.1. α-Tubulin

**Figure 3:**
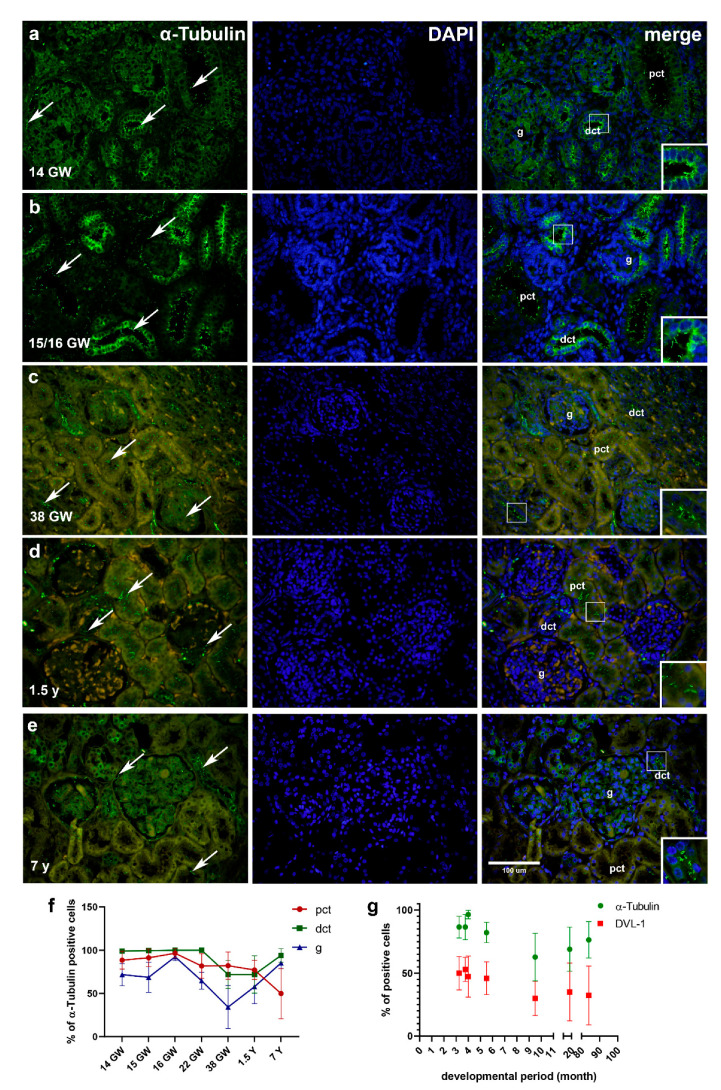
Immunofluorescence staining of α-tubulin and DAPI in the developing and postnatal human kidney tissues (**a**–**e**). Kidneys of 14th, 15th/16th, 38th GW (**a**–**c**); kidneys of 1.5- and 7-year-old children (**d**,**e**). Positive staining of α-tubulin (arrows) is shown in each structure in the cortex through developmental and postnatal phases (**a**–**e**), proximal convoluted tubules (pct), distal convoluted tubules (dct) and glomeruli (**g**). Details of primary cilia in pct (**d**) and dct (**a**–**c**,**e**) are shown as higher magnification insets (white box). Magnification ×40, scale bar 100 µm. The distribution of α-Tubulin positive cells per structure throughout different stages of development and in the postnatal period is shown in graph (**f**). Graph (**g**) (overall number of protein positive cells in observed structures) shows protein expression related to developmental time (Linear regression) and interrelation of α-Tubulin to DVL-1 through development and maturation (Two-Way ANOVA followed by SIDAK’s posthoc test). Data are shown as mean ± SD.

During development, α-tubulin is strongly expressed in all developing kidney structures, primarily visualizing the form of primary cilia on the surfaces of parietal epithelial cells of the Bowman’s capsule, g, pct and dct. Within kidney tissue, 82–97% of pct cells express α-tubulin, while in dct expression drops from 99% to 72% in the 38th GW (see Figure 3, Table 1). Among different developmental stages, the strongest expression of α-tubulin is found in the g of the 16th GW kidneys, containing 93% of positive cells. In the 14th, 15th and 22nd GW (see Figure 3a,b, Table 1), around 70% of the g cells express α-tubulin, while the least expression is observed in the 38th GW (*p* < 0.01) with 34% of the positive cells (Figure 3c). In the postnatal kidney tissue (1.5-years and 7-years old kidneys), the strongest immunoreactivity to α-tubulin is observed on the apical cell surface of the pct and dct (Figure 3d–f). The α-tubulin expression is also present in the perinuclear cytoplasm of the dct and the parietal epithelial cells of Bowman’s capsule. α-tubulin stains all renal structures with a mean of 50% of positive cells in pct, 94% in dct and 85% in g (Figure 3f). Dct stains significantly different from pct (*p* < 0.0001). All investigated structures stain positive to α-tubulin, with 77% of positive cells in pct, 72% in dct and 58% in g (Figure 3f).

The distribution of α-tubulin-positive cells per structure throughout different stages of development and in the postnatal period is shown in graph (f). Graph (g) (overall number of protein-positive cells in observed structures) shows protein expression related to developmental time (Linear regression) and interrelation of α-tubulin to DVL-1 through development and maturation (two-way ANOVA followed by Sidak’s post hoc test). Data are shown as means ± SD.

#### 2.2.2. Double Immunofluorescence Staining to Inversin and DVL-1 and DAPI Nuclear Stain in Developing and Healthy Postnatal Kidneys

##### Inversin Expression in Developing and Postnatal Kidney Tissue

**Figure 4:**
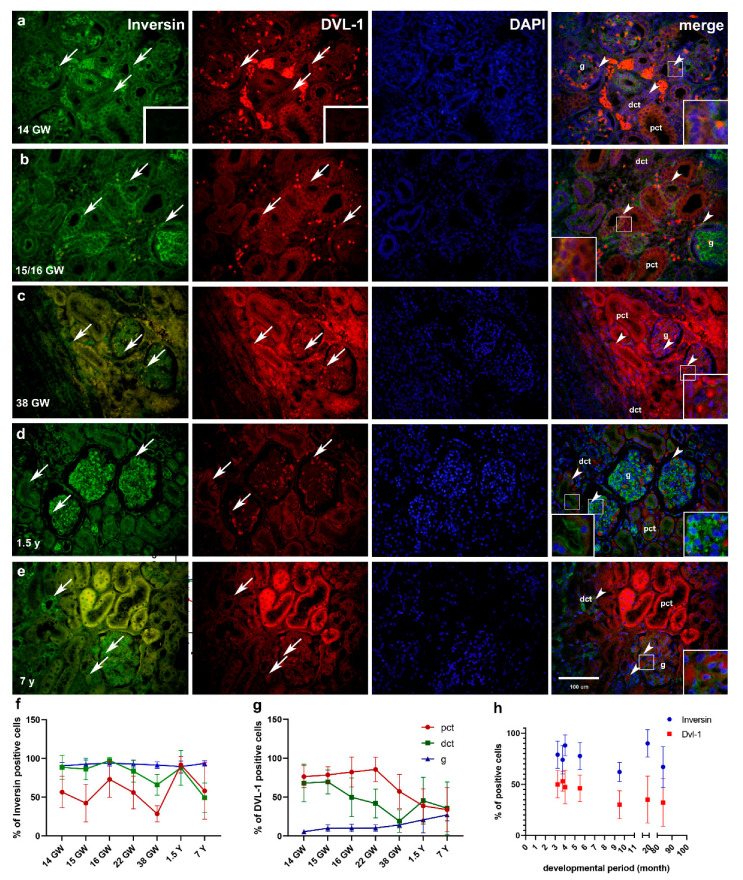
Double immunofluorescence staining of inversin (green), DVL-1 (red) and DAPI (blue) in the developing and postnatal kidney tissues (14th–38th GW, 1.5- and 7-year-old kidney tissues). Positive staining (arrows) is shown in each structure throughout all phases of development (**a**–**c**) and the postnatal period (**d**,**e**). Merged microphotos along with structures of interest in cortex: proximal convoluted tubules (pct), distal convoluted tubules (dct) and glomeruli (**g**). Co-localization of inversin/DVL-1 (arrowhead) is shown in merged microphotographs. Negative control stainings are shown as insets on inversin and DVL-1 (**a**). Erythrocytes can be seen as strongly stained cells near dct. Details are shown as higher magnification insets. Magnification ×40, scale bar 100 µm. Dynamic positive cell distribution of inversin and DVL-1 in kidney structures (pct, dct, g) throughout developing and postnatal stages are shown in graphs (**f**,**g**). Graph (**h**) shows protein expression (overall number of positive cells in structures) related to developmental time (Linear regression) and interrelation of inversin to DVL-1 through development and maturation (two-way ANOVA followed by Sidak’s post hoc test). Data are shown as means ± SD.

In the 14th, 15th and 16th GW, inversin shows strong granular expression in g, and mild granular expression in the cytoplasm of pct and dct (Figure 4a,b), while in the 22nd and 38th GW (see Figure 4c), the strongly positive expression is observed in g (Table 1). Between the 14th and 22nd GW, around 50% of the cells express inversin. In the 16th GW, pct cells express inversin in 73% of cells, while decrease to 29% of the cells (*p* < 0.05) in the 38th GW (see Figure 4f). Positive expression of inversin is found in approximately 80–90% of the dct and g cells in fetal kidney tissues of the 14th and 16th GW, with the lowest expression in dct of 38th GW (*p* < 0.05, *p* < 0.0001, respectively) where 66% of the cells were positive. In postnatal kidneys, pct stains strongly at the apical cell membrane, while dct stains mostly at the basal cell membrane and in the perinuclear cytoplasm (Figure 4d,e). At that stage, we found expression of inversin in all investigated renal structures, including pct, dct and g, with a mean expression of positive cells of 92% for pct, 88% for dct and 90% for g (Figure 4f). We did not observe a significant difference in signal strength of inversin expression between structures. The highest expression of inversin is found in g, with a mean of 94% of positive cells (Figure 4f). A significant difference is found comparing the staining of g (where 94% of cells stain positively) to pct, where 58% of cells stain positively (*p* < 0.01) and to dct, where 49% of cells are positive (*p* < 0.0001, Figure 4f).

##### DVL-1 Expression in Developing and Postnatal Kidney Tissue

Mild to strong expression of DVL-1 is observed in the cytoplasm of pct, dct and g in the fetal period (see Figure 4a–c, Table 1). In pct, 76–86% of the cells express DVL-1 in the 14th–22nd GW, while in the 38th GW, there are 57% of positive cells (Figure 4g). In the dct of 14th and 15th GW, 68–70% of the cells express DVL-1, in the 16th and 22nd GW, the number of positive cells drops to 42–50%, while in the 38th GW expression (*p* < 0.001), is observed in 19% of dct cells. Expression of DVL-1 increases in g throughout fetal stages: in the 14th GW, it is 6%, while in the 15th, 16th and 22nd GW, it increases to 10% (*p* < 0.01). In the 38 GW, 14% of the g cells express DVL-1 (Figure 4 g).

In the postnatal period, the DVL-1 immunoreactivity is dispersed in the cytoplasm (Table 1) of both pct and dct (Figure 4d,e). The signal is found in all the investigated structures but mostly in the perinuclear cytoplasm. The intensity of DVL-1 expression is significantly higher in pct compared to dct and g (*p* < 0.01). 39–45% of the cells in the pct and dct express DVL-1, while in g, 21% of the cells are positive (Figure 4 g). We did not find a significant difference between the percentage of immunoreactive cells in different renal structures. The percentage of DVL-1 immunoreactive cells is 34% in pct, 36% in dct and 27% in g, respectively (Figure 4g).

Co-expression of inversin and DVL-1 is observed in the cytoplasm of kidney g, dct and pct during development (see Figure 4a–c, merging). In the postnatal period, co-expression of inversin and DVL-1 characterizes different cellular compartments of tubular cells in pct and dct, while co-expression in g is characterized by the strong prevalence of inversin expression (see Figure 4d, merge). In the 7-year postnatal kidneys, co-expression of inversin and DVL-1 is seen in tubular cells of dct and pct, and in g where inversin expression slightly prevails in comparison to DVL-1 (Figure 4e, merge).

#### 2.2.3. Differences in Expression of α-Tubulin, Inversin and DVL-1 between Different Developmental Kidney Stages and Postnatal Kidneys

The number of α-tubulin-positive cells in pct of fetal kidney tissue was statistically significantly higher in comparison to the 7-year-old children’s kidney tissue (13th (*p* < 0.001), 15th (*p* < 0.0001) and 16th (*p* < 0.00001) GW). Dct of early fetal stages (13th, 15th, 16th and 22nd GW) had more positive cells in comparison to the 38th GW and 1.5-year-old children kidney tissue (*p* < 0.0001, respectively, Figure 3f).

When different developmental stages were compared, we found lower levels of inversin expression in the pct of the 13th (*p* < 0.0001), 15th (*p* < 0.00001), 22nd (*p* < 0.001) and 38th (*p* < 0.0001) GW in comparison to the renal tissue from the 1.5-year-old child. In dct, statistically significant lower expression of inversin was found comparing 16th GW to 38th GW kidney tissue (*p* < 0.0001). Furthermore, lower expression of inversin in dct was observed in 7-year-old child kidney tissue; comparing 13th, 15th (*p* < 0.001, both), 16th and 1.5-year-old kidney tissue to dct of 7-year-old kidney tissue (*p* < 0.00001, respectively, Figure 4f).

The observed signal of DVL-1 expression through the different stages revealed that pct of the 13th (*p* < 0.01), 15th, 16th (*p*< 0.001, respectively) and 22nd (*p* < 0.0001) GW had higher expression of immunoreactive cells comparing to the pct of 7-year-old children kidney tissue. DVL-1 immune-expression in dct of the kidney from 13th and 15th GW was significantly higher than in the 38th GW tissue (*p* < 0.0001, respectively, Figure 4g).

#### 2.2.4. Relationship between Expressions of α-Tubulin and Inversin to DVL-1 in Developing and Postnatal Kidney Tissue

α-Tubulin and inversin expressions were compared to DVL-1 expression throughout developmental stages and in postnatal kidney tissue. α-tubulin had statistically higher expression in all observed stages, comparing to the expression of DVL-1 (*p* < 0.001, Figure 3g). Through all the observed stages, inversin had a higher expression when compared to DVL-1 (*p* < 0.0001, Figure 4 h).

#### 2.2.5. Comparison of Immunohistochemical Staining to α-Tubulin, Inversin and DVL-1- and Statistical Analysis of Pathologically Changed Kidney Tissue (MCDK, CNF, FSGS)

##### α-Tubulin

**Figure 5:**
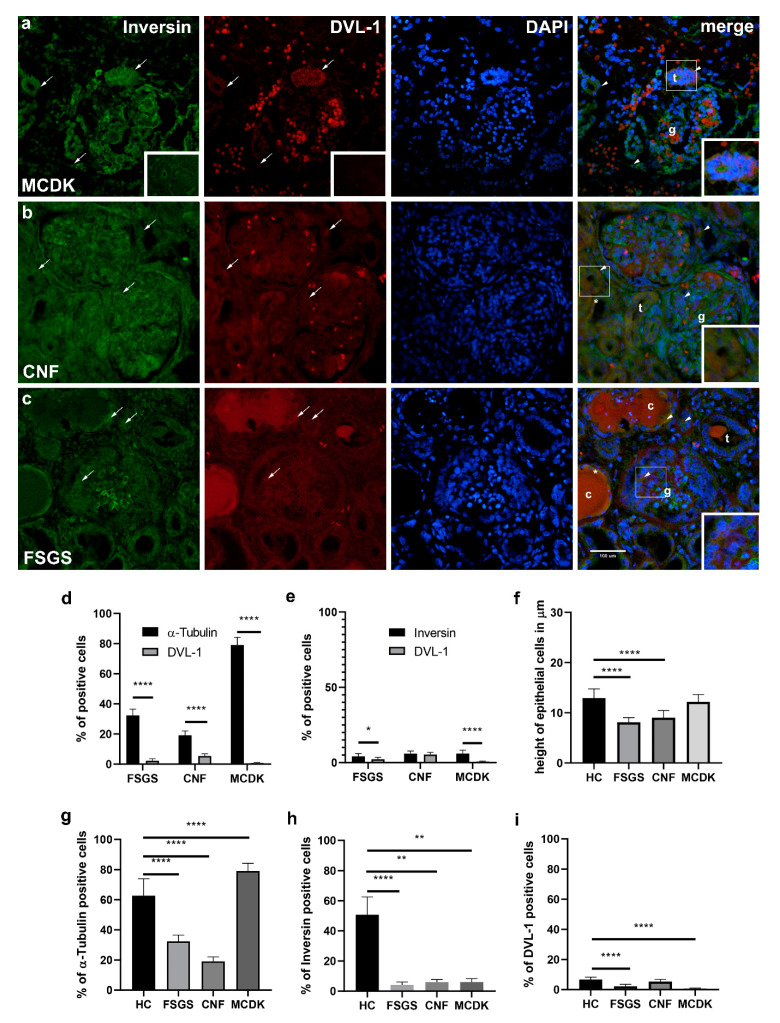
Double immunofluorescence staining of inversin (green), DVL-1 (red) and DAPI (blue) in pathological kidney tissues in MCDK (**a**), CNF (**b**) and FSGS (**c**): tubules (t), glomeruli (g), pct cyst (c), the height of pct epithelial cells (*). Structure and cell co-localization of inversin and DVL-1 (arrowheads) are shown in the merged sections with details in higher magnification insets. Negative control stainings are shown as insets on inversin and DVL-1 (a). Magnification ×40, scale bar 100 µm. Relationship of α-tubulin (**d**) and inversin (**e**) to DVL-1 expression in MCDK, FSGS and CNF (two-way ANOVA followed by SIDAK’s post hoc test). The difference in epithelial cell height (**f**) of FSGS, CNF and MCDK pct compared to healthy control (one-way ANOVA followed by Tukey’s post hoc test, *n* = 50). The difference in overall positive cells percentage of α-tubulin, inversin and DVL-1 in MCDK, CNF and FSGS when compared to healthy control (**g**–**i**), one-way ANOVA followed by Tukey’s post hoc test). Data are shown as means ± SD. Significant differences are indicated by *p*-value (* *p* < 0.05, ** *p* < 0.01, **** *p* < 0.0001).

The difference of α-tubulin staining in MCDK, FSGS and CNF was statistically significant in comparison to healthy postnatal kidney tissue as a control group (*p* < 0.0001, respectively). Dysplastic kidney tissues had significantly higher expression compared to the control group, while FSGS and CNF showed significantly lower expression of α-tubulin when compared to healthy control (Figure 5f).

##### Inversin

Inversin is expressed in the cytoplasm of disorganized epithelial cells and tubules of MCDK (Figure 5a). In CNF, inversin expression characterizes g and dct, while it shows less intensively in pct cysts (Figure 5b). In FSGS, inversin is strongly expressed in g and dct but less intensively in pct cysts (Figure 5c). Spatial expression of inversin showed a statistically significant higher staining rate in healthy kidney tissues (Figure 5g) when compared to MCDK, FSGS and CNF (*p* < 0.0001, respectively).

##### DVL-1

DVL-1 is very mildly expressed only in disorganized tubules of MCDK, while in CNF, its expression characterizes dct and g (Figure 5a,b). In FSGS, DVL-1 is observed as mild reactivity in g, dct and pct (Figure 5c). Healthy kidney tissues stained to DVL-1 showed a significantly higher rate of staining (Figure 5 h) in contrast to MCDK and to FSGS (*p* < 0.0001, both), while no significant difference was found for CNF.

#### 2.2.6. Relationship between Expressions of α-Tubulin and Inversin to DVL-1 in Pathologically Changed Kidney Tissue (MCDK, CNF, FSGS)

In MCDK, FSGS and CNF, α-tubulin is stained significantly higher than DVL-1 (*p* < 0.0001, respectively, Figure 5d). Inversin stained significantly higher when compared to DVL-1 in MDCK (*p* < 0.0001) and in FSGS (*p* < 0.01, Figure 5e).

#### 2.2.7. Differences in Epithelial Cell Height of Proximal Convoluted Tubules between Healthy Control and Pathologically Changed Kidney Tissue (MCDK, CNF, FSGS)

Height of pct epithelial cells (*n* = 50 per group) was compared between tubule cells in healthy kidney tissues and pathologically changed kidney tissues (Figure 5b,c,f). Mean epithelial cell height in HC was 12.91 µm ± 1.847 µm and was significantly higher when compared to CNF and FSGS (*p* < 0.0001, respectively). Mean cell height in CNF pct was 9.011 µm ± 1.453 µm, while in FSGS was 8.114 µm ± 0.9248 µm. Pct epithelial cells of MCDK did not show significant changes in height (12.17 µm ± 1.476 µm) when compared to HC.

#### 2.2.8. Differences in Primary Cilia Length between Healthy Control and Pathologically Changed Kidney Tissue (MCDK, CNF, FSGS)

**Figure 6:**
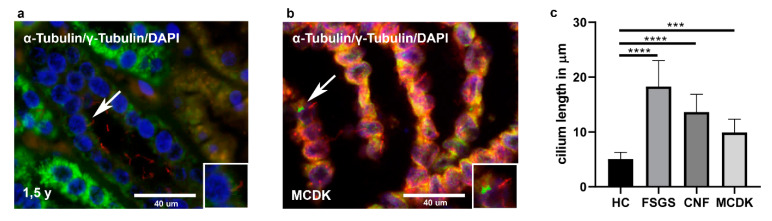
Double immunofluorescence staining with α-tubulin (red), γ-tubulin (green) and DAPI of the healthy 1.5-year-old kidney tissue (**a**) and in MCDK tissue (**b**). Arrows indicate tubulin. Details of primary cilia are shown as higher magnification insets. Graph showing differences in cilium length (*n* = 50, one-way ANOVA followed by Tukey’s post hoc test) between healthy control and pathological kidney tissues (**c**). Data are shown as means ± SD. *** *p* < 0.001, **** *p* < 0.0001).

Primary cilium length (*n* = 50 per group) was compared between HC and pathologically changed kidney tissues (Figure 6c). Healthy kidney tissues and MCDK were stained with γ-tubulin to reassure the specificity of α-tubulin cilium staining (Figure 6a,b). In healthy kidney tissues, primary cilium was 5.065 µm ± 1.229 µm long, while in MCDK, CNF and FSGS were significantly longer (*p* < 0.0005, respectively). Primary cilium length in MCDK was 9.908 µm ± 2.434 µm, in CNF 13.65 µm ± 3.218 µm, while in FSGS was significantly longer, 18.29 µm ± 4.717 µm, when compared to HC (*p* < 0.0001) and other pathological kidney tissues of MCDK (*p* < 0.0001) and CNF (*p* < 0.001).

## 3. Discussion

The aim of our study was to investigate an immunohistochemical expression of α-tubulin, inversin and DVL-1 in fetal renal tissue and postnatal kidney tissue. Furthermore, we wanted to explore whether the expression and staining pattern of α-tubulin, inversin and DVL-1 is disturbed in different kidney diseases when compared to healthy control. Namely, despite the extensive interest of the other researchers on the role of α-tubulin, inversin and DVL-1 during kidney development, most of the previous studies have used animals or in vitro experimental models. As far as we are aware, this is a first study that had shown expression and localization of α-tubulin along with inversin and DVL-1 in fetal and postnatal human kidneys, and that had explored the expression of mentioned proteins in kidney diseases, such as MCDK, FSGS and CNF. We also analyzed changes in cilia length and an appearance by light and electron microscopy, as our previous study has already indicated that ciliary disturbances may be associated with cystogenesis in FSGF and CNF [19].

In the canonical Wnt-signaling pathway, binding of Wnt ligands to receptors recruits Dvl [37]. Processes of the MET and the planar cell polarity pathway during the early stages of kidney morphogenesis are controlled by the Wnt-signaling. By mediating the Wnt pathway, the primary cilia control cell proliferation, differentiation and tissue morphogenesis [12] through organizing the cell cytoskeleton and cell orientation as well as tubular structure elongation [2,38]. In our study, α-tubulin, inversin and DVL-1 were all present in kidney structures, and all colocalized not only during fetal kidney development but also in the kidney tissue from the 1.5- and 7-year-old children. These findings are in agreement with the activation of the Wnt-signaling pathway that was proven to occur already during tubulogenesis [39]. Additionally, our results have revealed that the expression of α-tubulin and inversin are reciprocally related to DVL-1 and that they show a statistically significant difference between their expression patterns. That is in correlation to experimental models, as the mutation of the full Dvl protein family in mice results in a lack of gastrulation process, while mutations that do not include the whole protein family show defects in nodal cilia placement along with organ defects [40]. Previous findings suggested that inversin has a role in cell migration in *Xenopus* pronephros. Since that segment correlates to the mammalian loop of Henle and distal tubules, this could indicate the importance of inversin during kidney development in humans as well [41]. In agreement with that premise, previous studies disclosed that mutation of the inversin gene could result in nephronophthisis type 2, which is mediated by abnormal DVL-1 expression [42]. Despite the role of primary cilium being controversial in the regulation of the Wnt-signaling pathway, we found that α-tubulin colocalized with inversin and DVL-1 in the analyzed kidney samples. Furthermore, previous studies pointed out that the primary cilium along with inversin regulates the degradation of Dvl by influencing the Wnt-signaling pathway [43]. In human kidney diseases associated with the development of cysts, abnormal localization and function of primary cilia have been observed [19]. Similarly, the present study also confirmed morphological changes of primary cilia formation in pathological conditions such as CNF, FSGS and MCDK. Previous findings acknowledged that over-activation of canonical Wnt pathway following kidney damage leads to irreversible structural changes of kidney tissue [44]. On the contrary, primary cilium was given the role of switching from canonical to noncanonical Wnt pathway in assisting renal repair. Furthermore, over-activation of canonical Wnt pathway in transplanted kidney tissue was found to have a positive predictive value toward kidney fibrosis [45]. If canonical Wnt pathway prevails at the expense of noncanonical, it supports the theory of kidney fibrosis as the outcome. Our findings of lowered α-tubulin and inversin expression in CNF and FSGS imply inactivity of the noncanonical Wnt pathway, especially as both conditions tend to lead to end-stage renal disease. Namely, it is believed that normal localization and function of primary cilium could be a factor in maintaining a regular Wnt pathway and, therefore, a prerequisite for normal development. In contrast, if the Wnt pathway is enhanced, it can result in dysregulated cell proliferation and differentiation leading to carcinogenesis [46]. As previously described, the canonical Wnt pathway is mandatory for the initiation of MET and formation of the nephron, while its disturbance might lead to renal hypodysplasia [47]. Our results support this idea by revealing the lowest expression of DVL-1 with the highest expression of α-tubulin in MCDK in comparison to healthy control. These results may imply the lowest activation of the canonical Wnt pathway, which could be an underlying cause for the absence of nephron formation. In contrast, the highest α-tubulin expression with the lowest DVL-1 expression might explain the findings of multiple cysts as there is no organized elongation and cell polarization guided by the noncanonical Wnt pathway. Previous studies had also shown that inversin inhibits the canonical Wnt-signaling pathway by targeting Dvl for degradation [26]. This step in their interaction was found to be necessary to inhibit canonical Wnt-signaling in the maintenance of normal tubular elongation and positioning. Inversin mutation results in upregulation of canonical Wnt-signaling, which subsequently provoked abnormal proliferation in tubular cells. This step has been proven to be crucial in cystogenesis [42], while knockout of inversin in mice leads to polycystic kidney disease [48]. According to the theory of renal cystogenesis, inversin is considered a “cystoprotein”, because of its localization to primary cilia in renal tubular cells. In our study, primary cilia were present on the apical cell surface of tubular cells in all the analyzed stages, while during fetal stages, expression of α-tubulin was stronger than in the postnatal period.

Previous studies have shown that primary cilia function may be affected by dysregulation of α-tubulin during development, which may lead to cystogenesis, abnormal kidney development and possible chronic kidney disease in childhood [31,49]. This is in accordance with our findings of shortened and dysmorphic primary cilia observed in MCDK or with multiple and extremely elongated or dislocated cilia found in dilated tubules of CNF and FSGS when compared to healthy control. To support the fact that the canonical and noncanonical Wnt-signaling pathways, regulated by primary cilia, are considered to be mandatory for normal kidney development, we found significantly lower staining of inversin and DVL-1 in MCDK when compared to healthy control [31,49].

Different expression pattern dynamics of α-tubulin, inversin and DVL-1 throughout different kidney development phases may indicate switching between the canonical and noncanonical Wnt-signaling pathway during normal kidney morphogenesis. We suggest that their interchange determine transcription in canonical pathway or order of cell migration and polarization during kidney development in a noncanonical signaling pathway. Their balance and expression in all investigated kidney structures imply their important role in normal kidney development. During normal kidney development (from 13th GW to 38th GW), the overall expression of α-tubulin, inversin and DVL-1 is decreasing as kidney tissue acquires mature morphology. In 1.5- and 7-year-old kidney tissues, α-tubulin and inversin showed a slight increase in expression what supports the fact that the noncanonical Wnt pathway remains active after birth. On the contrary, DVL-1 persists with decreased expression pattern what might add to a conclusion that the canonical Wnt pathway is silenced in healthy kidney tissue. As a result of pathological kidney conditions, epithelial cells of the kidney might react with the reappearance of EMT and fibrosis [50] associated with reactivation of the canonical Wnt pathway [44]. Therefore, disturbances of α-tubulin, inversin and DVL-1 found in diseased kidneys might be the underlying pathological mechanism and a result of the switch from noncanonical to canonical Wnt pathway in the developing kidney alluding to the switch between reversible to irreversible kidney damage. Furthermore, changes in their prenatal and postnatal renal expression pattern might be associated with impairment of kidney function in adulthood, leading to congenital diseases and chronic kidney failure.

## 4. Materials and Methods

### 4.1. Human Samples

Samples of fetal kidney tissue were collected after pregnancy loss at the 14th, 15th, 16th, 22nd and 38th GW at the Department of Gynecology and Obstetrics of University Hospital Center Split. All acquired fetal material was examined by a pathologist, and only tissues without signs of abnormalities, macerations or intrauterine death and with normal karyogram were used for the study. Medical records of mothers were examined prior to sample collecting, and in case of health issues that might influence pregnancy outcome, kidney tissues were excluded from the study. Maturity was determined by head circumference, abdominal circumference and femur length [51] in correlation with the menstrual calendars of patients. Specimens of 1.5- and 7-year-old kidney tissue were collected after accidental deaths. The samples were obtained at the Department of Pathology of University Hospital Centre Split. The samples of the multicystic dysplastic kidney tissues (MCDK) were obtained after pregnancy loss, focal segmental glomerulosclerosis (FSGS) and nephrotic syndrome of the Finnish type (CNF) were obtained due to nephrectomy. All acquired material was examined and evaluated by a pathologist, who classified the diagnoses. The study protocol was approved by the Ethics Committee of the University of Split School of Medicine (20 May 2016) in accordance with the Helsinki Declaration and its updates (classification no.: 003-08/16-03/0001, registry no: 2181-198-03-04-16-0024, 20 May 2016) [52].

### 4.2. Immunohistochemistry

Collected tissue sample fixation was processed with 4% paraformaldehyde in phosphate-buffered saline (PBS) for 24 h at 22 °C. After dehydration in 100% ethanol, samples were embedded in paraffin, as described previously [53]. Specimens were sliced into 5 µm thick sections using a microtome and subsequently mounted on microscope slides. To verify tissue preservation, every 10th section was stained with hematoxylin and eosin [54]. Deparaffinization and immunohistochemistry were performed as previously described [2,55,56]. After rinsing in PBS, microscope slides were incubated overnight with primary antibodies in a humid chamber at 22 °C (StainTray slide staining system; Sigma-Aldrich, St. Louis, MO, USA). Primary antibodies used were rabbit monoclonal anti-alpha tubulin antibody (dilution 1:1000; ab179484, Abcam, Cambridge, UK), Rabbit Polyclonal Anti-inversin antibody (dilution 1:100; ab65187, Abcam, Cambridge, UK), mouse monoclonal DVL-1 antibody (1:150 dilution; sc-8025, Santa Cruz Biotechnology, Dallas, TX, USA) and rabbit polyclonal anti-gamma tubulin antibody (in order to verify primary cilia-specific staining with alpha-tubulin, 1:500 dilution; ab11321, Abcam, Cambridge, UK). After rinsing in PBS, secondary antibodies were administered for an hour as listed: donkey anti-rabbit IgG H&L, Alexa Fluor 488 (dilution 1:400; ab150073, Abcam, Cambridge, UK) and goat anti-mouse IgG H&L, TRITC (dilution 1:400; ab6786, Abcam, Cambridge, UK) 4’,6-diamidino-2-phenylindole dihydrochloride (DAPI) was used for staining of the nuclei and after a 2-min incubation, slides were washed in PBS and overlaid with mounting medium and coverslips. Non-specific staining was prevented by using protein block (ab64226; Abcam, Cambridge, UK) before the primary antibody application. As a negative control, a pre-adsorption test was conducted, while the specificity of the secondary antibodies was checked by omitting the primary antibodies from the staining procedures.

### 4.3. Electron Microscopy

Fixation of kidney tissues was performed in 4% paraformaldehyde for 24 h, followed by post-fixation in 1% osmium tetroxide for 1 h. The dehydration process was performed with ethanol series and finished with embedding in LX 112 resin [22]. One-micrometer-thin sections were stained using toluidine blue and studied to select ultrathin sections. Ultrathin sections with a thickness of 0.05 micrometers were examined after staining with uranyl acetate and lead citrate. JEOL 1200 EX microscope was used to obtain the microphotographs.

### 4.4. Genetic Analysis

As previously described [19], using leukocytes from the peripheral blood, genomic DNA was extracted. A homozygous missense mutation in the NPHS1 gene was found (c.1096A > C; p.Ser366Arg) what confirmed the diagnosis of congenital nephrotic syndrome of the Finnish type (CNF) [57].

### 4.5. Data Analysis

The section analysis was performed on a fluorescence microscope using 3 fluorescence channels (Olympus BX51, Tokyo, Japan). Images were captured by digital camera DP71 (Olympus, Tokyo, Japan) on high-power (×40) magnification. Only the renal cortex containing proximal convoluted tubules (pct), distal convoluted tubules (dct) and glomeruli (g) were of interest. Images were then processed by ImageJ software (Rasband, W.S., ImageJ, U.S. National Institutes of Health, Bethesda, MD, USA, https://imagej.nih.gov/ij/ (accessed on 15 October 2018), 1997–2021.) and Adobe Photoshop (Adobe Inc., San Jose, California, USA) for further evaluation. Prior to cell counting, the split channels ImageJ tool was used. Afterward, the original immunofluorescence microscopic photo was subtracted by the red or green channel (depending on what the original channel was) using the image calculator ImageJ tool to prevent signal leakage. Values under the threshold level 50 were considered negative. We counted immunoreactive signals in cells of at least 20 structures (pct, dct or g) per stage or overall positive number of cells per micro photo of multicystic dysplastic kidney tissue, FSGS and CNF. We classified cells as positive if the immunofluorescence signal accumulated at any level of the membrane, cytoplasm or nucleus above value 50 measured on ImageJ software using threshold command. Negative cells were categorized as cells with the absence of any immunoreactivity. Two independent investigators analyzed the data.

### 4.6. Semiquantitative Analysis

The intensity of α-tubulin, inversin and DVL-1 staining signal was evaluated by two independent researchers using ImageJ software. Overall signal intensity was measured after the image was set in 8-bit type; afterward, the intensity of marker staining was evaluated in 20 pct, dct and g by measuring outlined area of the structure. If results between evaluators were different, a third independent researcher clarified the doubt. Maximal signal intensity for α-tubulin was 84.125 ± 3.214 SD, for inversin was 71.50 ± 2.715 SD and for DVL-1 80.916 ± 1.875 SD. The highest saturation of signal color on the microscope photographs was marked as 3 (66.66–99.99% of overall signal image intensity), followed by 2 (33.33–66.66%) for the intermediate intensity of signal and 1 (0.33%–33.33%) for low signal intensity (Table 1.).

### 4.7. Statistical Analysis

For the determination of differences between stages and structures, the Kruskal–Wallis test was done, followed by Dunn’s post hoc using GraphPad Prism software (Graphpad Software Inc., San Diego California, USA, www.graphpad.com (accessed on 15th of October 2018.)). The number of positive cells was expressed in percentage as mean value ± standard deviation (SD), while statistical significance was acknowledged at *p* < 0.05. The number of analyzed structures was 4200 in 35 samples, with an overall number of 135,256 cells counted.

We used 2-way ANOVA with Sidak’s post hoc test to test differences in expression of α-tubulin, inversin and DVL-1 in different developmental stages. To study protein expression concerning developmental time, we used Linear regression.

One-way ANOVA followed by Tukey’s post hoc test was used to explore the differences in expression of α-tubulin, inversin and DVL-1 in healthy kidney tissue compared to tissues of MCDK, FSGS and CNF. Differences in expression of proteins between MCDK, FSGS and CNF were investigated with 2-way ANOVA followed by Sidak’s post hoc test.

One-way ANOVA followed by Tukey’s post hoc test was used to evaluate the differences in cilia length and epithelial cell height of pct between healthy control and pathological kidney tissues. Data were shown as mean value ± standard deviation (SD) with statistical difference acknowledged at *p* < 0.05.

## Figures and Tables

**Table 1:** Semiquantitative staining intensity to α-tubulin, inversin and dishevelled-1 (DVL-1) during kidney developing stages and 1.5- and 7-year-old kidney tissues. In each group, 20 structures of interest were observed. * g—glomeruli, pct—proximal convoluted tubules, dct—distal convoluted tubules, GW—gestational week, y—years of postnatal development.

Antibody	α-Tubulin							Inversin							DVL-1						
GW/ystructure *	14	15	16	21	38	1.5	7	14	15	16	21	38	1.5	7	14	15	16	21	38	1.5	7
Pct	3	3	3	3	2	2	2	1	1	1	1	1	2	1	1	1	1	1	3	2	1
Dct	3	3	3	3	3	3	3	2	2	2	2	2	2	3	1	2	2	2	3	1	1
G	2	1	2	3	1	2	2	2	3	3	3	3	3	3	1	1	1	3	3	3	3

Number 3 demonstrates strong reactivity (for α-tubulin signal measure 56.083–84.117; for inversin 47.667–71.492; for DVL-1 53.944–80.908); 2 demonstrates moderate reactivity (for α-tubulin 28.042–56.082; for inversin 23.833–47.666; for DVL-1 26.972–53.993); 1 demonstrates mild reactivity (for α-tubulin 0.278–28.041; inversin 0.246–23.832; DVL-1 0.267–26.971).

## Abbreviations

DVL-1	Dishevelled-1
GW	Gestational week
MET	Mesenchymal to epithelial transition
CKD	Chronic kidney disease
MCDK	Multicystic dysplastic kidneys
FSGS	Focal segmental glomerulosclerosis
CNF	Nephrotic syndrome of the Finnish type
pct	Proximal convoluted tubules
dct	Distal convoluted tubules
g	Glomeruli
